# Kabuki syndrome: a Chinese case series and systematic review of the spectrum of mutations

**DOI:** 10.1186/s12881-015-0171-4

**Published:** 2015-04-21

**Authors:** Shuang Liu, Xiafei Hong, Cheng Shen, Quan Shi, Jian Wang, Feng Xiong, Zhengqing Qiu

**Affiliations:** Department of Pediatrics, Peking Union Medical College Hospital, Chinese Academy of Medical Sciences and Peking Union Medical College, Beijing, China; Peking Union Medical College, Beijing, China; BGI research, Shenzhen, China; Endocrinology Department, Children’s hospital of ChongQing Medical University, ChongQing, China

**Keywords:** Kabuki syndrome, *KMT2D*, Chinese, Series, Dandy-Walker syndrome, Spinal bifida

## Abstract

**Background:**

Kabuki syndrome is a rare hereditary disease affecting multiple organs. The causative genes identified to date are *KMT2D* and *KDMA6*. The aim of this study is to evaluate the clinical manifestations and the spectrum of mutations of *KMT2D*.

**Methods:**

We retrospectively retrieved a series of eight patients from two hospitals in China and conducted Sanger sequencing for all of the patients and their parents if available. We also reviewed the literature and plotted the mutation spectrum of *KMT2D*.

**Results:**

The patients generally presented with typical clinical manifestations as previously reported in other countries. Uncommon symptoms included spinal bifida and Dandy-Walker malformation. With respect to the mutations, five mutations were found in five patients, including two frameshift indels, one nonsense mutation and two missense mutations.

**Conclusions:**

This is the first case series on Kabuki syndrome in Mainland China. Unusual symptoms, such as spinal bifida and Dandy-Walker syndrome, suggested that neurological developmental defects may accompany Kabuki syndrome. This case series helps broaden the mutation spectrum of Kabuki syndrome and adds information regarding the manifestations of Kabuki syndrome.

## Background

Kabuki syndrome or Kabuki make-up syndrome was originally described in 1981 following observations of five Japanese children whose conditions were characterized by mental retardation, dwarfism and peculiar faces and abnormal dermatoglyphics [1]. Historically, the diagnostic criteria for Kabuki syndrome were mainly based on the typical clinical manifestations, which were established by analyzing a group of sixty-two patients with Kabuki syndrome in 1988 [2]. Five cardinal manifestations were frequently observed in patients with Kabuki syndrome and could be used as diagnostic clues, including peculiar facial appearances, mild-to-moderate mental retardation, dermatoglyphic abnormalities, skeletal anomalies, and postnatal growth deficiencies. Kabuki syndrome was previously considered to be prevalent in only Japan, but it has now been recognized to be prevalent all over the world. The estimated prevalence in Japan is approximately 1/32,000, whereas the estimated prevalence is at least 1/86,000 in Australia and New Zealand [2,3].

The underlying genetic mutation of Kabuki syndrome was not revealed until the year 2010, when exome sequencing identified *MLL2* mutations in Kabuki syndrome patients (Kabuki syndrome 1, OMIM 147920) [4]. Bögershausen et al proposed a new nomenclature for the *MLL2* gene as *KMT2D* [5]. *KMT2D* consists of fifty-four coding regions and functions as a histone-lysine N-methyltransferase in various signalling pathways such as epigenetic modulating. However, *KMT2D* mutations alone were not able to account for all Kabuki syndrome cases. Later, mutations in the *KDM6A* gene, which encodes a histone demethylase that interacts with *KMT2D*, were identified (Kabuki syndrome 2, OMIM 300867) [6]. Therefore, analyzing mutations in the *KMT2D* and *KDM6A* genes would help confirm the diagnosis in patients who fulfilled the clinical diagnostic criteria for Kabuki syndrome.

Medical centres and institutions in Europe, North America and South America have carried out extensive *KMT2D* mutation spectrum reports. However, studies concerning the *KMT2D* mutation spectrum in China have rarely been reported. This study describes the first case series of eight Chinese patients with Kabuki syndrome, their clinical manifestations, and their atypical symptoms. We also analyzed the genetic changes in the *KMT2D* gene and conducted a literature review of the *KMT2D* mutation spectrum. The main aim of this study was to determine the mutation spectrum of the *KMT2D* gene in Chinese Kabuki patients and to review the mutation spectrum reported in the literature.

## Methods

### Patients

This study was reviewed and approved by the Peking Union Medical Collage Hospital Ethics Review Board. All of the participants’ legal guardians provided their written informed consents to participate on behalf of the children and/or for themselves. We obtained written informed consent to publish characteristic features of the disease and to maximally hide other non-disease-related features, thus protecting the privacy for each patient.

We retrospectively searched the clinical records for the initial outpatient visit covering from January 2010 to February 2013 in the Department of Pediatrics, Peking Union Medical College Hospital (PUMCH), Beijing, China and Children’s Hospital of ChongQing Medical University (CH-CQMU), ChongQing, China. Those patients with a clinical diagnosis of “Kabuki syndrome” were enrolled. Overall, eight patients were enrolled. Clinical manifestations were retrieved from the original clinical records. A telephone-based follow-up was conducted to ask for patients’ current height and weight as of February 2013.

### Sanger sequencing of *KMT2D* gene and identification of pathogenic mutations

*KMT2D* gene mutation status was obtained by Sanger sequencing after obtaining informed consent. First, peripheral blood samples from each patient were collected. Blood samples from the parents were also collected if available. Then, the total genomic DNA was extracted by standard procedures, and the 54 exons and exon-intron junctions of the *KMT2D* gene (UCSC NM_003482) were amplified in three PCR fragments. Exons were amplified in a reaction containing 2 μL of 10x PCR buffer, 3 μL of dNTPs (2.5 mmol/L), 0.3 μL of rTaq polymerase (5 U/μL) (TAKARA, Dalian, China), 1 μL of genomic DNA (100 ng/μL), 1 μL of each primer (10 pmol/μL), and 11.7 μL of ddH2O. The thermal cycler conditions were as follows: 95°C for 5 min, then 35 cycles of 94°C for 30 s, 59°C for 30 s, and 72°C for 45 s, and a final elongation step at 72°C for 5 min. Direct sequencing was performed on a Genetic Analyzer (Biomed Corp, Beijing, China). As a reference, the A of the ATG translation initiation codon of coding sequence of *KMT2D* was referred to as nucleotide +1.

Results from Sanger sequencing were compared with the reference sequence of *KMT2D* to identify single nucleotide substitutions, frameshift indels, and non-frameshift indels. Novel missense mutations would be subjected to further analysis to explore their pathogenicity. First, the parents’ *KMT2D* genes were sequenced. Second, the proband’s missense mutation was searched in the 1000 Genomes Database and in the Exome Variant Server [7]. Finally, the pathogenicity of protein change due to the missense mutation was predicted using the in silico prediction models SIFT, PROVEAN and Polyphen-2 [8,9].

### Literature review of the *KMT2D* gene mutation spectrum

Two of the authors (CS and XH) independently searched the published literature in MEDLINE and EMBASE using the following search keys: (“Kabuki syndrome” OR “Kabuki make-up syndrome” OR “Niikawa-Kuroki syndrome”) AND (“*KMT2D*” OR “*MLL2*”), without language restriction, with published data up to January 6, 2015. Another author (ZQ) supervised the literature review process and resolved any disagreement between the authors (CS and XH) regarding a study’s inclusion. Articles were manually reviewed, and *KMT2D* mutation data were retrieved. Here, we included only studies with 10 or more patients. The *KMT2D* mutations were summarized and categorized as “missense,” “frameshift indel” or “nonsense.”

## Results

### Clinical manifestations of eight Chinese patients with Kabuki syndrome

We identified eight patients with a clinical diagnosis of Kabuki syndrome (six from PUMCH and two from CH-CQMU). Six patients were male, and two were female. The ages of initial diagnosis ranged from 8 months to 9 years. All of patients belonged to the Han ethnic group.

**Figure 1:**
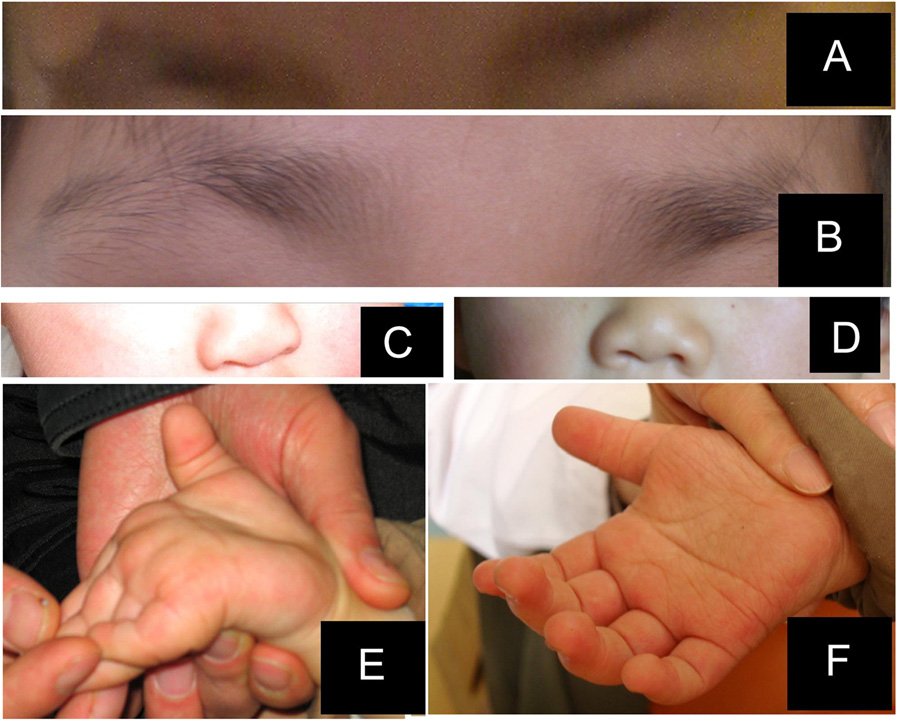
Typical patient abnormalitie. **(A)** High/sparse eyebrows by Patient 2. **(B)** High/sparse eyebrows demonstrated by Patient 3. **(C)** Depressed nasal tip demonstrated by Patient 5. **(D)** Depressed nasal tip demonstrated by Patient 8. **(E)** Hyperlaxity demonstrated by Patient 8. **(F)** Fingertip pads demonstrated by Patient 8.

The general clinical manifestations were categorized in a “five cardinal manifestations” pattern, which included typical craniofacial abnormalities, skeletal abnormalities, dermatoglyphic abnormalities, cerebral abnormalities, and postnatal growth deficiencies (Table 1). The percentages of patients with each symptom were compared with previous reports [10]. The typical features of the aforementioned patients are illustrated in Figure 1.

**Table 1:** **Clinical manifestations of eight patients with Kabuki syndrome**

**Clinical manifestation**		**Patient 1**	**Patient 2**	**Patient 3**	**Patient 4**	**Patient 5**	**Patient 6**	**Patient 7**	**Patient 8**	**Percentage in this series**	**Cumulative percentage**
General Information	Gender	M	M	F	M	F	M	M	M		
	Age of diagnosis	7 y	3 y	8 y	8 mo	2 y	9 y	4 y 11 m	4 y 1 m		
	Age at follow-up	8 y	4 y			3 y			5 y 6 m		
Typical craniofacial abnormality	High/sparse eyebrows	+	+	+	+	+	+	+	+	100.0%	85%
	Long palpebral fissures	+	+	+	+	+	+	+	+	100.0%	99%
	Blue sclera	-	+	+	+	+		-	+	71.4%	31%
	Strabismus	-	-	-	-	-	-	+	-	12.5%	36%
	Ptosis	-	-	-	-	-	-	-	-	0.0%	50%
	Large ears	+	+	+	+	+	+	+	+	100.0%	84%
	Depressed nasal tip	+	+	+	+	+	+	+	+	100.0%	83%
	Micrognathia	-	+	-	+			+	+	66.7%	40%
	Abnormal dentition	-	-	+					+	50.0%	68%
skeletal abnormality	Clinodactyly of the 5th finger	+	+	-	+	-	-	-	+	50.0%	50%
	Hip dislocation	+	-	-	-	-	-		-	14.3%	18%
	Hyperlaxity	+	-	-	-	-	-		+	28.6%	74%
	Scoliosis/vertebral malformation	+	-	-	-	-	-	-	-	12.5%	32%
Dermatoglyphic abnormalities	Fingertip pads	+	+	+	+	+	+	+	+	100.0%	89%
Cerebral abnormality	Mental retardation	+	+	+	+	+	+	-	+	87.5%	84%
	Hypotonia	-	-	-	-	-	-	-	+	12.5%	68%
	Seizures	-	-	+	-	+	-	-	-	25%	17%
	Head circumference at initial outpatient diagnosis (cm)	51	46.7	50	42.5	42	49.8	49	46.7		
	Microcephaly	-	-	-	-	+	-	-	+	25%	26%
Postnatal growth	Weight at birth (g)	3250	2500	2650		2950			2000		
	Body length at birth (cm)	50		48		49					
	Weight at initial outpatient diagnosis (kg)	26	13		7		20	14	11		
	Height at initial outpatient diagnosis (cm)	121	101				107	99	86		
	Weight at follow-up (kg)	27	15			12					
	Height at follow-up (cm)	125	102			96			98		
	Postnatal growth retardation	-	-			-	+	+	-	33.3%	55%
	Feeding difficulties	+	-	-	-	-	-	-	-	12.5%	
Other common symptoms	Frequent infections	+	+	-	-	+	-	+	+	62.5%	60%
	Cardiac anomalies	-	-	-	-	+	-	+	+	37.5%	42%
Special features		+	+

All of the patients in this case series presented with typical craniofacial abnormalities, including high/sparse eyebrows, long palpebral fissures with eversion of the lateral third of the lower eyelids, large ears and depressed nasal tips and blue sclera. The percentages of patients presenting with strabismus and ptosis were lower than in previous reports.

With respect to skeletal abnormalities, four patients presented with clinodactyly of the 5th finger. Patient 1 was diagnosed with bilateral hip dislocation at one year of age and underwent corrective surgery. The same patient also presented with hyperlaxity and spina bifida occulta. Patient 8 also presented with hyperlaxity.

All of the patients presented with fingertip pads typical of previously reported dermatoglyphic abnormalities. With respect to cerebral abnormalities, seven out of the eight patients presented with mental retardation. Only Patient 5 and Patient 8 presented with microcephaly (two standard deviations from the median value) according to the standard for Chinese children [11]. With respect to seizures, Patient 5 and Patient 3 exhibited this manifestation. Patient 5 presented with an episode of seizure that occurred in the first day of life and was infantile spasm type. MRI indicated corpus callosum hypoplasia and Dandy-Walker malformation, which indicated that congenital cerebral developmental defects might happen concomitantly with Kabuki syndrome.

With respect to postnatal growth, Patient 6 and Patient 7 were diagnosed with postnatal growth retardation, and Patient 1 experienced self-reported feeding difficulties.

In addition to these five cardinal manifestations, Patient 1 experienced fever or diarrhoea every other week from the age of two to three. Patient 2 experienced multiple upper respiratory symptoms and tonsillitis when he was 3 years old. Patients 5, 7 and 8 experienced frequent respiratory infections. With respect to cardiac abnormalities, Patient 5 was diagnosed with a patent foramen ovale and Patient 7 and Patient 8 presented with atrial septal defects. Patient 2 presented with pectus carinatum, and Patient 3 presented with bilateral knee joint stiffness.

### Review of the *KMT2D* gene mutation spectrum in Kabuki syndrome

We performed a literature review of the published studies on *KMT2D* gene mutations based upon the method described above. The number of studies from MEDLINE and EMBASE were 48 and 182, respectively. After merging the duplicates, there were 201 published articles. Each abstract was manually read, and data were retrieved if available. The data from the included studies contributed to the further analysis.

Briefly, we included 12 studies for further analysis [4,12-22] to compare the distribution of the previously reported *KMT2D* gene mutations with our case series. We first summarized the different types of mutations from these studies.

**Figure 2:**
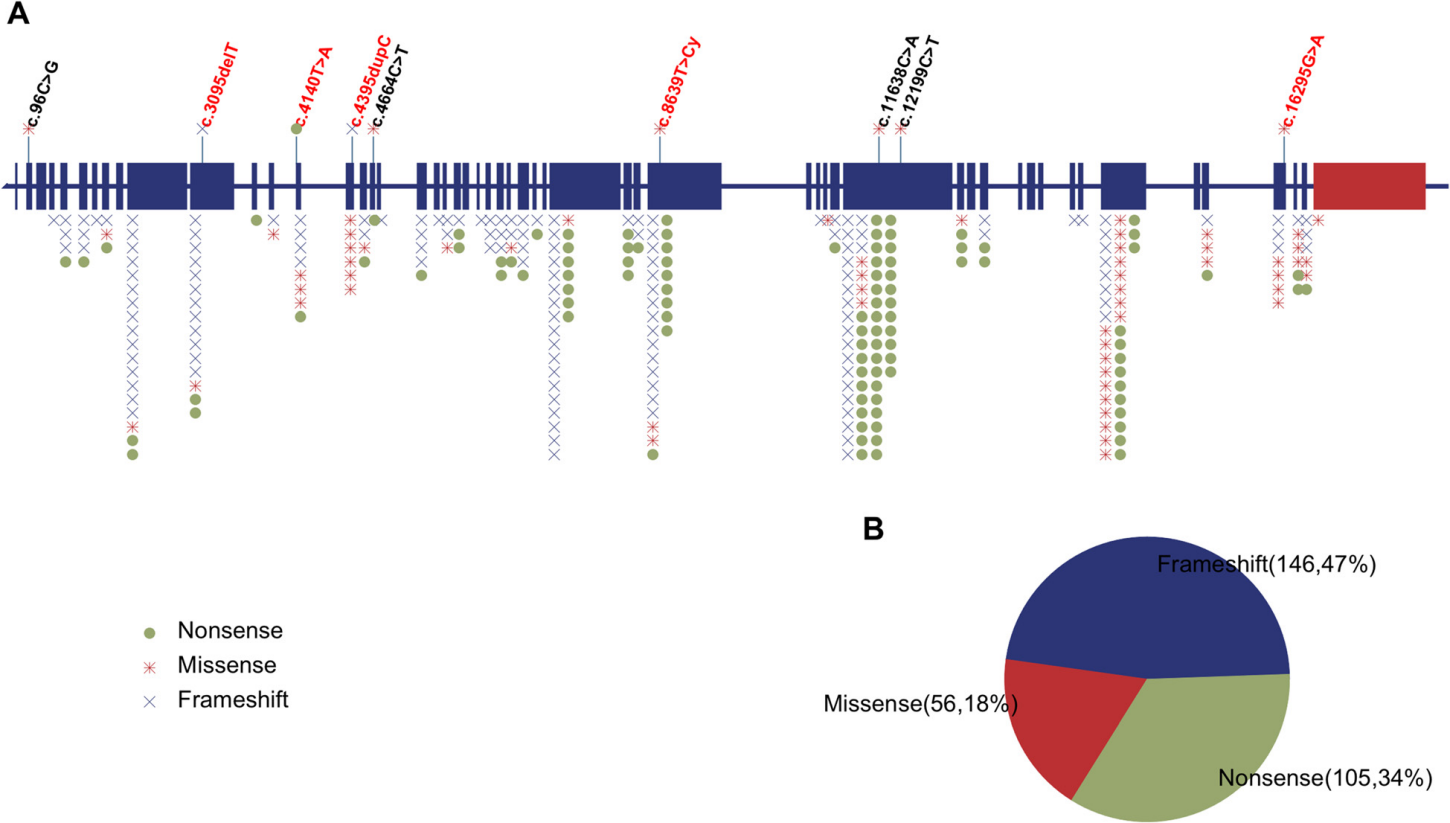
Mutation spectrum of the *KMT2D* gene. **(A)** Schematic view of the *KMT2D* gene. Each note above represents a variant in this case series (a red note indicates a pathogenic mutation). Each type of mutation is illustrated as a dot with a distinct combination of colour and shape. Each dot below represents a genetic mutation reported in the literature. **(B)** Schematic view of the number and proportion of missense, nonsense and in-frame indel mutations in the *KMT2D* gene.

There were 146 (47%) frameshift indels, 105 (34%) nonsense mutations, and 56 (18%) pathogenic missense mutations (Figure 2). We did not include splice-site and in-frame indels because the pathogenicity of these mutations would require specific investigation. Mutation hotspots were identified in exons 10, 31, 34, 39, and 48.

### Sanger sequencing to identify mutations in the *KMT2D* gene in this study

**Figure 3:**
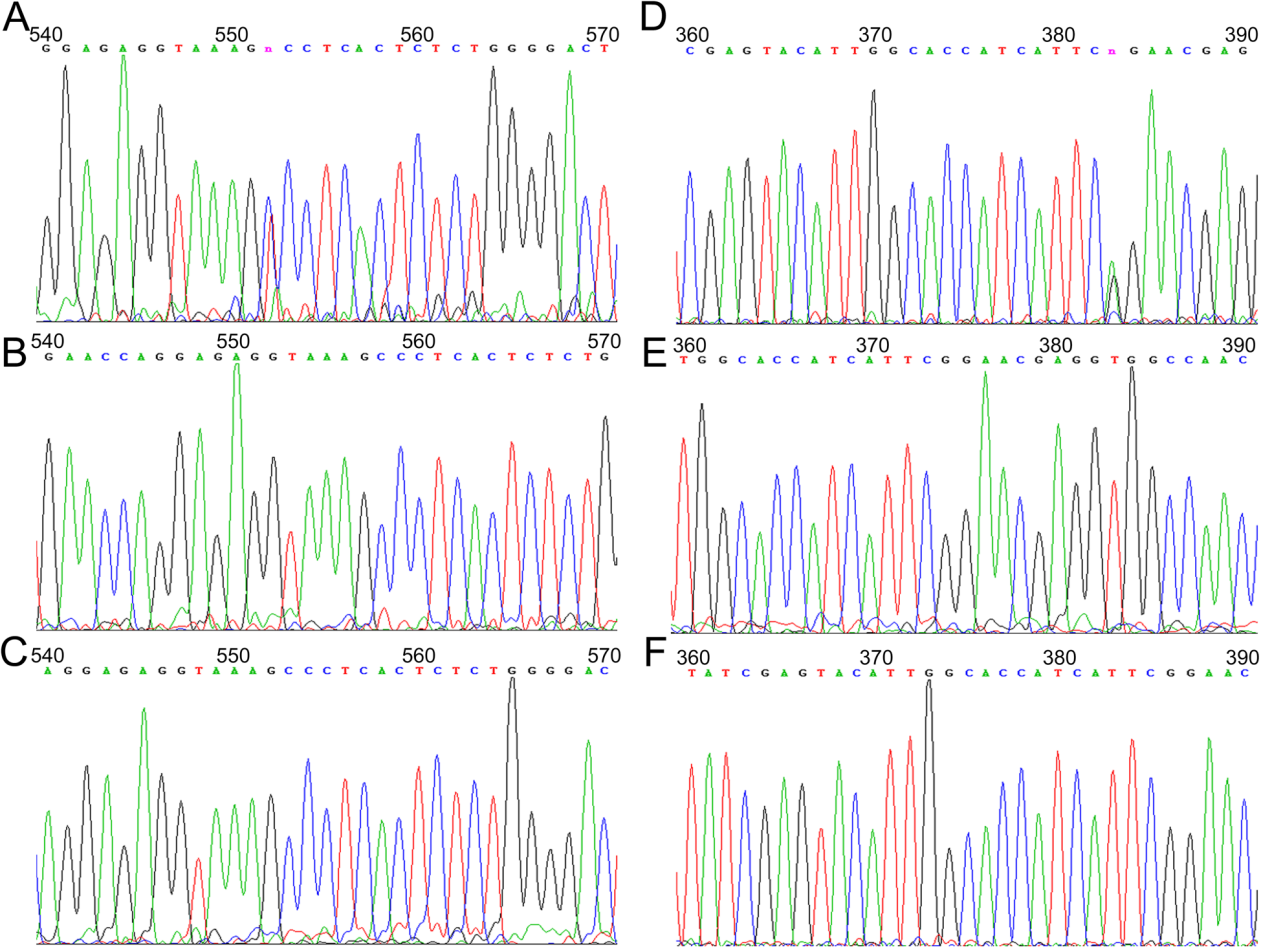
Sanger sequencing of Patient 1 with two de novo mutations. **(A)** The missense mutation c.12199C > T in Patient 1. **(B, C)** Both parents had no mutations at c.12199. **(D)** The missense mutation c.16295G > A in Patient 1. **(E, F)** Both parents had no mutation at c. 16295.

Our eight patients underwent genetic analysis of the *KMT2D* gene, and ten variants were detected (Table 2). Unlike the distribution in previous reports, only two frameshift indels and one nonsense mutation were identified, which were c.3095delT in Patient 4, c.4395dupC in Patient 6 and c.4140T > A in Patient 8. One in-frame indel, c.11718-11723delGCAACA, was also identified in Patient 8. Six missense variants were also identified. Patient 1 had two missense variants, c.12199C > T and c.16295G > A (Figure 3). Patients 2, 3, 5 and 7 had one missense variant each: c.4664C > T, c.8639T > C, c.96C > G (p.Asp32Glu) and c.11638C > A, respectively. We sequenced both of the parents of Patients 1, 2, 3 and 5 and found none of the previously described missense variants in the parents’ *KMT2D* genes. The other four patients’ parents were not available for genetic testing.

**Table 2:** ***KMT2D*** **gene variants analysis**

**Patient**	**Mutation**	**Type of mutation**	**Predicted protein changes**	**Inheritance**	**Exon**	**PROVEAN prediction**		**SIFT prediction**		**Polyphen-2 prediction**		**Pathogenicity**	**Novelty**
						**Score**	Prediction*	**Score**	Prediction**	**Score**	**Prediction**		
Patient 1	c.12199C > T	Missense	p.Pro4067Ser	De novo	39	−1.663	Neutral	0	Damaging	0.085	Benign	Undetermined	Novel
	c.16295G > A	Missense	p.Arg5432Gln	De novo	51	−3.695	Deleterious	0	Damaging	1.000	Probably damaging	Confirmed	Kokitsu-Nakata et al
Patient 2	c.4664C > T	Missense	p.Ser1555Phe	De novo	17	−0.958	Neutral	0.682	Tolerated	0.976	Probably damaging	Undetermined	Novel
Patient 3	c.8639T > C	Missense	p.Leu2880Pro	De novo	34	−5.055	Deleterious	0	Damaging	1.000	Probably damaging	Confirmed	Novel
Patient 4	c.3095delT	Frameshift indel	p.Leu1032Argfs24X	N/A	11							Confirmed	Novel
Patient 5	c.96C > G	Missense	p.Asp32Glu	De novo	2	0.423	Neutral	0.106	Tolerated	0.001	Benign	Undetermined	Novel
Patient 6	c.4395dupC	Frameshift indel	p.Lys1466Glnfs25X	N/A	15							Confirmed	Novel
Patient 7	c.11638C > A	Missense	p.Leu3880Met	N/A	39	0.500	Neutral	0.008	Damaging	0.003	Benign	Undetermined	Novel
Patient 8	c.4140T > A	Nonsense	p.Cys1370X	N/A	14							Confirmed	Novel
	c.11718-11723delGCAACA	Non-frameshift indel	p3907-3909delQQ	N/A	39	−1.298	Neutral					Undetermined	Novel

*Cutoff = -2.5.

**Cutoff = 0.05f.

We did not find any of the missense variants identified in this series in the 1000 Genomes Database or the Exome Variant Server.

With respect to the pathogenicity analysis, we used the in silico prediction models SIFT, PROVEAN and Polyphen-2 to predict the protein changes due to the missense variants. Two missense variants (c.16295G > A and c.8639T > C) were predicted to be pathogenic by all three of the models. Therefore, we categorized those two variants as “pathogenicity confirmed.” However, the other four missense variants (c.12199C > T, c.4664C > T, c.96C > G and c.11638C > A) were predicted to be benign, neutral or tolerable by at least two of the programs. Therefore, we would categorize those four variants as “pathogenicity undetermined.” The single in-frame indel in Patient 8 (c.11718-11723delGCAACA) was also categorized as “pathogenicity undetermined.”

In conclusion, five of the eight patients had pathogenic mutations in the *KMT2D* gene. These patients were Patient 1 (c.16295G > A), Patient 3 (c.8639T > C), Patient 4 (c.3095delT), Patient 6 (c.4395dupC), and Patient 8 (c.4140T > A). Three of the eight patients did not have confirmatory pathogenic mutations in the *KMT2D* gene, and the clinical symptoms could not be ascribed to *KMT2D* gene mutations. These patients were Patient 2, Patient 5, and Patient 7.

Nine of the ten variants were novel. One missense variant (c.16295G > A) was previously reported by Kokitsu-Nakata et al [23].

## Discussion

Kabuki syndrome is a rare congenital disease. Cases from different parts of the world have been extensively reported. However, reports on Chinese patients with Kabuki syndrome have been rare. To our knowledge, five cases of Chinese patients due to this disease were published in several case reports [24-27]. However, the *KMT2D* mutation status was not available for these cases. This is the first case series to include both the typical clinical manifestations and the *KMT2D* mutation status of patients from Mainland China. The clinical manifestations in this case series were consistent with the clinical diagnostic criteria.

With respect to atypical symptoms, Patient 5 presented with hypoplasia of the corpus callosum and Dandy-Walker malformation. Increasing evidence suggests that structural central nervous system (CNS) malformations, including Dandy-Walker malformation, can be present in Kabuki syndrome patients [28-31]. These congenital neurologic defects may partially account for the mental retardation commonly observed in Kabuki syndrome patients. Further analysis is needed to evaluate whether CNS structural defect are frequent in patients with Kabuki syndrome.

With respect to congenital heart defects, the major types associated with Kabuki syndrome are left-sided obstructions and aortic dilation, coarctation of the aorta (COA), atrial septal defects (ASDs), ventral septal defects (VSDs), and tetralogy of Fallot (TOF), among others [32,33]. In our series, two patients presented with ASDs. Thus, we propose that patients should be meticulously screened for congenital heart defects once the diagnosis of Kabuki syndrome has been made.

Gene sequencing of *KMT2D* and *KDM6A* can be used to detect mutations in patients with a clinical diagnosis of Kabuki syndrome. Studies with large cohorts of patients indicate that *KMT2D* gene mutations can be detected in half to three-fourths of patients with clinical diagnoses of Kabuki syndrome [13,16,17]. Pathogenic *KDM6A* gene mutations account for a small percentage of patients [14,16,19,34-36].

*KMT2D* pathogenic mutations were detected in five of the eight patients in our case series. This case series identified six missense variants, two frameshift indels, one in-frame indel and one nonsense mutation. All of the frameshift indels and nonsense mutations were considered pathogenic because the protein structure was significantly altered. However, we would urge caution in interpreting the influence of missense mutations or in-frame indels on protein function because some amino acid substitutions would not necessarily impair protein function. Therefore, we used in silico prediction models to analyze the missense mutations and non-frameshift indels.

We found two patients with two variants in their *KMT2D* genes. Patient 1 had two missense mutations (c.12199C > T and c.16295G > A), which were both absent from the parents’ *KMT2D* genes, indicating that they were de novo mutations. Both mutations were absent from the 1000 Genomes Database and the Exome Variant Server, indicating that both mutations are rare in the general population. One mutation, c.12199C > T, was predicted to be pathogenic by the SIFT program but not by PROVEAN or Polyphen-2. The other mutation, c.16295G > A, was predicted to be pathogenic by all three in silico models and has been reported by Kokitsu-Nakata et al in a Brazilian case of familial Kabuki syndrome [23]. Therefore, we considered c.16295G > A to be pathogenic, whereas the pathogenicity of c.12199C > T was inconclusive and could potentially be non-pathogenic.

Patient 8 also had two variants. We could not determine the inheritance status because the parents of Patient 8 refused to give blood samples for sequencing of the *KMT2D* gene. One mutation was a nonsense mutation (c.4140T > A), which was considered to be pathogenic. The other mutation in this patient was an in-frame deletion (c.11718-11723delGCAACA), which was predicted to be non-pathogenic by the PROVEAN prediction model. Therefore, the pathogenicity of c.11718-11723delGCAACA was inconclusive and could potentially be non-pathogenic. To our knowledge, it is a rare event for a patient to carry two mutant variants of the *KMT2D* gene. A previous study by Hannibal et al reported only three patients who carried two *KMT2D* gene variants in a cohort of 110 patients [22].

## Conclusion

This report is the first case series of Kabuki syndrome patients with definitive genetic diagnosis in China. These data help broaden the mutation spectrum of Kabuki syndrome and add information regarding the manifestations of Kabuki syndrome.
